# Ceralasertib Monotherapy in Patients with ATM-Altered Advanced Solid Tumors or Metastatic Castration-Resistant Prostate Cancer: Data from the Phase IIa PLANETTE Study

**DOI:** 10.1158/2767-9764.CRC-26-0184

**Published:** 2026-07-02

**Authors:** Rahul Aggarwal, Antoine Italiano, Susan M. Domchek, Oscar Goodman, Sophie Postel-Vinay, Jesus Garcia-Donas, Tanya Dorff, Zachery R. Reichert, Armelle Vinceneux, Neal Shore, Catherine H. Marshall, Joshua Armenia, Graeme Parr, Aleksandra Kmieciak, Natalia Lukashchuk, Olga Murina, Daniel Slade, Neel Shah, Bienvenu Loembé, Emma Dean, Elhan Sanai, Wassim Abida

**Affiliations:** 1 Division of Hematology/Oncology, University of California, San Francisco, California.; 2 Department of Medicine, Institut Bergonié, Bordeaux, France.; 3 Faculty of Medicine, University of Bordeaux, Bordeaux, France.; 4 Basser Center for BRCA, University of Pennsylvania, Philadelphia, Pennsylvania.; 5 Hematology/Oncology, Nevada Oncology Specialists, Las Vegas, Nevada.; 6 Drug Development Department, U981 INSERM Research Unit, Institut Gustave Roussy, Villejuif, France.; 7 Cancer Institute, University College of London, London, United Kingdom.; 8 Gynecologic and Genitourinary Tumors, HM Hospitales – Centro Integral Oncológico HM Clara Campal, Madrid, Spain.; 9 Genitourinary Disease Program, City of Hope Comprehensive Cancer Center, Duarte, California.; 10 University of Michigan, Rogel Comprehensive Cancer Center, Ann Arbor, Michigan.; 11 Medical Oncology, Centre Léon Bérard, Lyon, France.; 12 START Carolinas/Carolina Urologic Research Center, Myrtle Beach, South Carolina.; 13 Oncology, Johns Hopkins University, Baltimore, Maryland.; 14 Oncology Data Science, AstraZeneca, Cambridge, United Kingdom.; 15 Oncology Early Development Clinical, AstraZeneca, Cambridge, United Kingdom.; 16 Clinical Pharmacology and Quantitative Pharmacology, AstraZeneca, Cambridge, United Kingdom.; 17 Translational Medicine, AstraZeneca, Cambridge, United Kingdom.; 18 Statistical Science, AstraZeneca, Cambridge, United Kingdom.; 19 Oncology Early Development Clinical, AstraZeneca, Waltham, Massachusetts.; 20 Oncology Early Development, AstraZeneca, Cambridge, United Kingdom.; 21 Genitourinary Oncology Service, Memorial Sloan Kettering Cancer Center, New York, New York.

## Abstract

**Purpose:**

Ceralasertib is an oral ataxia telangiectasia and Rad3–related (ATR) inhibitor with preclinical activity in ataxia-telangiectasia mutated (ATM)-altered cancers. The phase IIa PLANETTE study evaluated ceralasertib in previously treated advanced solid tumors (cohort A) or metastatic castration-resistant prostate cancer (cohort B) with ATM alterations (*ATM* mutations and/or ATM protein deficiency).

**Patients and Methods:**

Patients received ceralasertib 160 mg twice daily on days 1 to 14 of a 28-day cycle. Efficacy was evaluated in patients with centrally confirmed ATM alterations. The primary endpoint was objective response rate (ORR; cohort A) or composite response rate (CRR: radiographic response, prostate-specific antigen response, or circulating tumor cell conversion; cohort B). Secondary endpoints included progression-free survival (PFS) and safety.

**Results:**

Cohorts A and B included 30 and 15 patients, respectively. ORR (cohort A) was 7.1% [80% confidence interval (CI), 1.9–17.9; *n* = 2/28]: one complete response ongoing at 14.1 months (breast cancer) and one partial response ongoing at 7.4 months (endometrial cancer). CRR (cohort B) was 7.7% (80% CI, 0.8–28.6; *n* = 1/13). In patients with centrally confirmed ATM protein loss, the ORR was 18.2% (80% CI, 4.9–41.5; *n* = 2/11) and CRR was 0% (80% CI, 0–28; *n* = 0/7). The median PFS was 3.7 months in each cohort (cohort A, 80% CI, 1.9–5.6; cohort B, 80% CI, 1.9–not calculable). Grade ≥3 adverse events (AE) occurred in 50% of cohort A and 53.3% of cohort B patients. The most common AEs overall were asthenia/fatigue, nausea, and anemia.

**Conclusions:**

Ceralasertib monotherapy was tolerated; however, responses were limited. Alternative patient selection and combination treatments are being explored.

**Significance:**

In the phase IIa PLANETTE study in advanced/metastatic ATM-altered cancers, ceralasertib 160 mg twice daily (days 1–14, 28-day cycle) was tolerated; objective responses were limited, corroborating previous findings with ATR inhibitors in ATM-deficient tumors. Optimizing biomarker-based patient selection beyond ATM deficiency represents a key aspect of future ATR inhibitor development.

## Introduction

Ataxia-telangiectasia mutated (ATM) and ataxia telangiectasia and Rad3–related (ATR) are serine/threonine protein kinases that play interconnected roles in complementary DNA damage response (DDR) pathways to maintain genomic integrity (1–3). ATM is activated in response to DNA double-strand breaks (DSB) and induces G1/S cell-cycle arrest by stabilizing p53, whereas ATR prevents the formation of DSBs by inducing G2/M cell-cycle arrest following its recruitment to stalled DNA replication forks (1, 4). Inhibition of ATR leads to DSB accumulation and cell death in specific genomic contexts (5). This is particularly true in genomically unstable and DDR-deficient (e.g., ATM loss) tumor cells or tumors with high replication stress, suggesting that ATR inhibition alone may be a promising targeted therapy (6).

Complete loss of function (LoF) of the *ATM* gene (through deleterious and/or pathogenic mutations) or the absence of ATM protein expression is thought to increase cellular sensitivity to inhibitors of DDR, particularly ATR inhibitors (7). Thus, ATM LoF alterations may identify cancers that are sensitive to treatment with ATR inhibition (8). Both somatic and germline *ATM* mutations have been identified in solid and lymphoid malignancies, including breast, colorectal, lung, pancreatic, and prostate cancers (9, 10), with an overall prevalence of approximately 2% to 5.4% across most common tumor types (11). The highest prevalence of *ATM* mutations is reported for prostate cancer (11), with a prevalence ranging from 4% to 6% in metastatic castration-resistant prostate cancer (mCRPC; ref. 12), and >70% of patients with prostate cancer with *ATM* mutations have biallelic *ATM* LoF (11). It is important to note that not all pathogenic *ATM* mutations in tumors lead to protein loss, and conversely, some tumors with ATM protein loss may not harbor pathogenic *ATM* mutations (13–15). *ATM* mutations that confer clinical sensitivity to ATR inhibition have been difficult to define, and it remains to be established whether pathogenic biallelic mutations or protein loss would better predict response. Therefore, dual comparative assessment by next-generation sequencing (NGS) and immunohistochemistry (IHC) is warranted (2, 7).

Ceralasertib is a selective and potent oral ATR inhibitor that has demonstrated evidence of activity in ATM-defective cancers in preclinical (7, 16–19) and clinical (6, 20–23) investigations. For example, in the phase I OLAPCO study, five patients with relapsed or refractory cancers with *ATM* mutations received ceralasertib plus the poly-ADP ribose polymerase inhibitor (PARPi) olaparib. One patient with metastatic breast cancer harboring a germline *ATM* mutation and biallelic *ATM* LoF in their tumor experienced a durable complete response (CR) and another patient had stable disease (SD), both lasting >26 months (21). In the phase I PATRIOT study, ceralasertib monotherapy induced durable responses in patients with advanced solid tumors harboring DNA damage response defects such as loss of ARID1A (24). Other ATR inhibitors such as elimusertib (BAY1895344; refs. 14, 25) and camonsertib (RP-3500; refs. 15, 26) have also reported monotherapy activity in patients with *ATM* variants.

The phase IIa PLANETTE study (NCT04564027) was conducted to enable the accurate evaluation of ceralasertib monotherapy in patients with advanced/metastatic tumors with *ATM* mutations and/or ATM protein loss.

## Patients and Methods

### Study design and treatment

PLANETTE was an open-label, multicenter study in patients with advanced solid tumors harboring germline or somatic *ATM* mutations (including deleterious or suspected deleterious and pathogenic or likely pathogenic *ATM* gene variants). Eligible patients were enrolled into cohort A: advanced solid tumors excluding prostate cancer and non–small cell lung cancer (NSCLC), which was assessed in the HUDSON study (27), and cohort B: mCRPC.

Patients in both cohorts received ceralasertib 160 mg orally twice daily on days 1 to 14 of a 28-day cycle until disease progression or unacceptable toxicity. The first nine patients (eight in cohort A and one in cohort B) had started on ceralasertib 240 mg orally twice daily on days 1 to 14 of a 28-day cycle, but this dose was not tolerated due to a high rate of grade 3 to 4 cytopenias. Efficacy was not analyzed in patients who started on ceralasertib 240 mg twice daily due to the small sample size; efficacy and safety analyses did not include data for patients who started on ceralasertib 240 mg twice daily. The most common adverse events (AE) observed in patients receiving ceralasertib 240 mg in cohort A are shown in Supplementary Table S1.

The study was performed in accordance with the Declaration of Helsinki, the International Conference on Harmonization Good Clinical Practice guidelines, and all applicable laws and regulations. The protocol and all modifications were approved by relevant ethics committees and regulatory authorities. All patients provided written informed consent.

### Patients

Patients ≥18 years of age with histologically confirmed advanced solid tumors [except prostate cancer and NSCLC (cohort A)] or mCRPC (cohort B) who progressed on prior treatment, had no standard treatment options, and had an *ATM* mutation identified by a preexisting local tumor or blood [germline or circulating tumor DNA (ctDNA)] test were enrolled to start the study treatment. Confirmation of an *ATM* mutation and/or ATM protein deficiency via a central test was required for inclusion in the efficacy analyses, regardless of the local test result prior to enrollment. Presence of *ATM* mutations was determined by NGS [FoundationOne CDx (F1CDx; Foundation Medicine, Inc. (FMI)) for tumors and FoundationOne Liquid CDx (F1LCDx) for ctDNA]. ATM protein deficiency was assessed using an IHC antibody for ATM (Y170; Roche Diagnostics) with a ≤5% expression cutoff.

Biallelic *ATM* LoF status was evaluated as an exploratory analysis based on tumor NGS using F1CDx according to criteria described previously (11). Germline *ATM* mutation status was evaluated as an exploratory analysis using Myriad MyRisk Hereditary Cancer Test and/or tumor NGS using F1CDx. *ATM* alterations originating from clonal hematopoiesis of indeterminate potential (CHIP) were not assessed.

Patients in cohort A had to have measurable disease per Response Evaluation Criteria in Solid Tumors version 1.1 (RECIST 1.1); patients in cohort B had to have tumors measurable by RECIST 1.1 and/or an unfavorable CellSearch circulating tumor cell (CTC) count (≥5 cells per 7.5 mL blood). Patients in cohort A must have experienced disease progression on previous treatment prior to study entry. Patients in cohort B must have experienced disease progression on ≥1 androgen receptor pathway inhibitor (ARPi) and ≥1 taxane regimen, unless refused by the patient or contraindicated, and had radiographic evidence of disease progression and/or prostate-specific antigen (PSA) progression, and castrate serum testosterone levels ≤50 ng/dL (≤1.75 nmol/L). Patients in both cohorts were required to have an Eastern Cooperative Oncology Group performance status of 0 to 2, normal organ and bone marrow function, and a life expectancy ≥16 weeks. Key exclusion criteria included previous therapy with an ATR inhibitor and symptomatic and/or uncontrolled brain metastases.

### Endpoints

The primary endpoint in cohort A was objective response rate (ORR) by investigator assessment per RECIST 1.1. The secondary endpoints in cohort A were duration of response, best percentage change in target lesion diameter, and progression-free survival (PFS; all investigator-assessed per RECIST 1.1), and safety.

The primary endpoint in cohort B was composite response rate (CRR), comprising any one of the following: radiologic response assessed by the investigator per RECIST 1.1 for soft tissue lesions, confirmed CellSearch CTC count conversion from unfavorable to favorable (≥5 cells per 7.5 mL blood to <5 cells per 7.5 mL blood), or confirmed PSA decline ≥50% in the absence of disease progression by RECIST 1.1 for soft tissue lesions. The secondary endpoints in cohort B included duration of radiologic response, best percentage change in target lesion diameter (both investigator-assessed), radiologic PFS [investigator-assessed per RECIST 1.1 for soft tissue lesions and Prostate Cancer Working Group 3 (PCWG3) criteria for bone lesions], and safety. Other secondary endpoints planned for cohort B (ORR per RECIST 1.1 and PCWG3 criteria and the proportions of patients with a confirmed CTC count conversion from unfavorable to favorable and PSA decline ≥50%) were not analyzed due to the small sample size following early termination of enrollment.

AEs were described using the Medical Dictionary for Regulatory Activities version 25.1 and graded according to Common Terminology Criteria for Adverse Events version 5.0. Pharmacokinetic parameters were assessed in both cohorts as an exploratory endpoint. Blood samples were collected on day 8 and day 14 before ceralasertib administration (pre dose) and 1 hour (±30 minutes) post dose. Ceralasertib concentrations in plasma were measured using a bioanalytical assay with a lower limit of quantification of 41.3 ng/mL and a quantification range of 41.3 to 41,300 ng/mL. Select endpoints were also examined in genetically defined *ATM* subgroups, including patients with biallelic *ATM* LoF, germline *ATM* mutations, and *ATM* mutations detected in ctDNA only.

### Statistical analyses

Planned enrollment was approximately 25 patients in cohort A and approximately 27 patients in cohort B, all with ATM alterations by central testing. The planned sample sizes were determined to provide adequate precision for associated confidence intervals (CI) when estimating the primary endpoints.

Primary endpoints were analyzed using a 2-sided 80% CI calculated via the Clopper–Pearson method. Patient demographic and safety data were summarized descriptively. Median PFS was evaluated using the Kaplan–Meier method, and associated CIs were calculated using a log–log (Greenwood) formula. Efficacy analyses were conducted in all patients who were molecularly eligible with ATM alterations by central testing, received ≥1 dose of ceralasertib, and had measurable baseline disease (cohorts A and B) and/or an unfavorable CTC count at baseline (cohort B only). Safety analyses were conducted in all patients who received ≥1 dose of ceralasertib. Statistical analyses were performed using SAS version 9.4 or higher (SAS Institute Inc.).

## Results

### Patients

**Figure 1.**
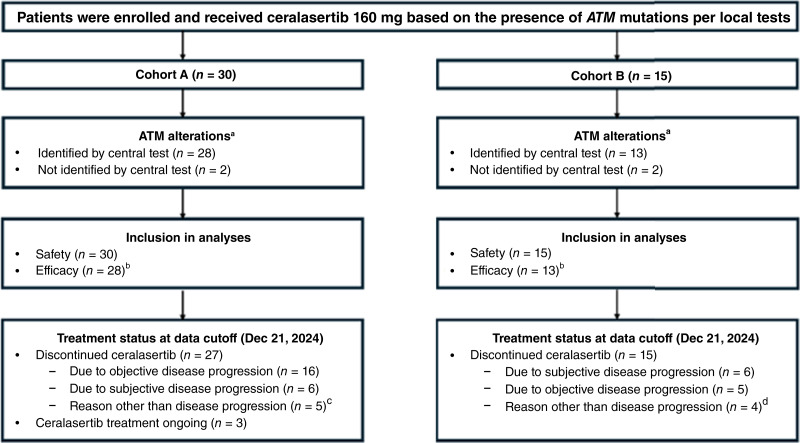
CONSORT diagram for patients who started on ceralasertib 160 mg twice daily. ^a^*ATM* mutations and/or ATM protein loss by IHC: four patients total (two in cohort A and two in cohort B) were enrolled based on local tests reporting *ATM* variant of unknown significance alterations, but central confirmation did not identify *ATM* mutations (tumor/ctDNA NGS) or ATM protein deficiency (tumor IHC). ^b^Per protocol, efficacy analyses were conducted in patients with ATM alterations (*ATM* mutations and/or ATM protein loss) identified by central testing. ^c^Due to AE, consent withdrawal, death, patient decision, and other (*n* = 1 each). ^d^Unknown (*n* = 3) or patient decision (*n* = 1).

In cohort A, 30 patients were enrolled and had been treated at the data cutoff (December 21, 2022), of whom 28 had ATM alterations (*ATM* mutations and/or ATM protein deficiency) by central testing and were evaluable for response. In cohort B, 15 patients were enrolled and had been treated at the data cutoff (April 28, 2023), of whom 13 had ATM alterations by central testing. A total of four patients (two from each cohort) did not undergo efficacy analysis because they did not have ATM alterations confirmed by central testing. Per protocol, the primary analysis for cohort A occurred approximately 6 months after 25 response-evaluable patients with ATM alterations by central testing were enrolled. Recruitment to cohort B was terminated early because of insufficient ceralasertib activity in the primary cohort A analysis and in a parallel *ad hoc* analysis of cohort B; the results of these analyses are presented for cohorts A (ORR) and B (CRR) below. Three patients in cohort A and none in cohort B were receiving ceralasertib at the data cutoffs. CONSORT diagrams for patients who started on ceralasertib 160 and 240 mg twice daily in both cohorts are shown in Fig. 1 and Supplementary Fig. S1, respectively.

The median age of patients was 66.5 years (range, 32–83) in cohort A and 72 years (range, 56–81) in cohort B (Table 1). Almost all patients (92.9% in cohort A and 100% in cohort B) had metastatic disease at study entry, and 28.6% of patients in cohort A and 53.8% of patients in cohort B had received ≥4 prior lines of systemic therapy. The most common cancer type in cohort A was colorectal cancer (25% of patients). Additional disease characteristics specific to cohort B are summarized in Supplementary Table S2. The representativeness of patients in this study versus the wider disease populations is discussed in Supplementary Table S3. The proportions of patients positive/negative/unknown for ATM protein–deficient tumors were 39.3%/42.9%/17.9% in cohort A and 53.8%/38.5%/7.7% in cohort B. The proportions of patients positive/negative/unknown for *ATM* mutations per central tumor and ctDNA NGS were 64.3%/14.3%/21.4% and 92.9%/3.6%/3.6%, respectively, in cohort A and 38.5%/23.1%/38.5% and 84.6%/15.4%/0%, respectively, in cohort B. The proportions of patients positive/negative/unknown for biallelic *ATM* LoF were 46.4%/7.1%/46.4% in cohort A and 15.4%/7.7%/76.9% in cohort B. The proportions of patients positive/negative/unknown for germline *ATM* mutations were 46.4%/14.3%/39.3% in cohort A and 38.5%/0%/61.5% in cohort B (Table 1). Further analyses regarding the prevalence of ATM alterations identified in tumor samples (by NGS or IHC) versus ctDNA only (NGS) are summarized in Supplementary Table S4.

**Table 1.** Demographics and baseline disease characteristics for patients with ATM alterations by central testing who started on ceralasertib 160 mg twice daily.

Parameter	Cohort A (*n* = 28)	Cohort B (*n* = 13)
Age, median (range), years	66.5 (32–83)	72 (56–81)
Sex, *n* (%)	​	​
Male	14 (50)	13 (100)
Female	14 (50)	0
Race, *n* (%)	​	​
White	9 (32.1)	6 (46.2)
Other	3 (10.7)	0
Not reported	16 (57.1)	7 (53.8)
ECOG PS, *n* (%)	​	​
0	11 (39.3)	3 (23.1)
1	16 (57.1)	7 (53.8)
2	1 (3.6)	3 (23.1)
Prior lines of systemic therapy, *n* (%)	​	​
1	4 (14.3)	1 (7.7)
2	10 (35.7)	2 (15.4)
3	6 (21.4)	3 (23.1)
≥4	8 (28.6)	7 (53.8)
Cancer type^a^, *n* (%)	​	​
Colorectal	7 (25)	0
Biliary tract	4 (14.3)	0
Pancreatic adenocarcinoma	4 (14.3)	0
Breast	2 (7.1)	0
Ovarian epithelial	2 (7.1)	0
Other^b^	9 (32.1)	0
mCRPC	0	13 (100)
Metastatic disease at study entry, *n* (%)	26 (92.9)	13 (100)
*ATM* gene status per central tumor NGS, *n* (%)	​	​
Mutant	18 (64.3)	5 (38.5)
Wild-type or VUS	4 (14.3)	3 (23.1)
Unknown	6 (21.4)	5 (38.5)
*ATM* gene status per central ctDNA NGS, *n* (%)	​	​
Mutant	26 (92.9)	11 (84.6)
Wild-type or VUS	1 (3.6)	2 (15.4)
Unknown	1 (3.6)	0
Biallelic *ATM* LoF, *n* (%)	​	​
Yes	13 (46.4)	2 (15.4)
No	2 (7.1)	1 (7.7)
Unknown	13 (46.4)	10 (76.9)
Germline *ATM* mutation, *n* (%)	​	​
Yes	13 (46.4)	5 (38.5)
No	4 (14.3)	0
Unknown	11 (39.3)	8 (61.5)
ATM protein expression by central IHC, *n* (%)	​	​
≤5%	11 (39.3)	7 (53.8)
>5%	12 (42.9)	5 (38.5)
Unknown	5 (17.9)	1 (7.7)

Abbreviations: ECOG PS, Eastern Cooperative Oncology Group performance status; VUS, variant of uncertain significance.

a Evaluated as a *post hoc* analysis.

b Adrenal, ampullary, bladder, endometrial, jejunum, leiomyosarcoma, melanoma, pancreatic neuroendocrine tumor, and perigastric cancers (*n* = 1 each).

### Disease response

Key efficacy outcomes for both cohorts are summarized in Table 2, and outcomes for genetic *ATM* subgroups are shown in Supplementary Table S5. In cohort A, the ORR among patients with ATM alterations by central testing (*n* = 28) was 7.1% (80% CI, 1.9–17.9) and the ORR both among patients with biallelic *ATM* LoF and among patients with germline *ATM* mutations (*n* = 13 each) was 15.4% (80% CI, 4.2–36). The two objective responses were ongoing at data cutoff: one CR ongoing at 14.1 months in a patient with breast cancer, and one partial response (PR; −56.9% tumor reduction) ongoing at 7.4 months in a patient with endometrial cancer (Table 3). Both patients with objective responses were germline carriers of pathogenic or likely pathogenic *ATM* variants and had ATM protein–deficient tumors with biallelic *ATM* LoF. Both also had pathogenic or likely pathogenic variants of a second DDR gene: a germline pathogenic *CHEK2* variant in the breast tumor and a pathogenic *ARID1A* variant in the endometrial tumor. Target lesion reduction in the responding patient with endometrial cancer is shown in Supplementary Fig. S2.

**Table 2.** Ceralasertib efficacy in patients with ATM alterations by central testing who started on ceralasertib 160 mg twice daily.

	All patients	ATM protein expression by IHC		
		≤5%	>5%	Unknown
Cohort A	(*n* = 28)	(*n* = 11)	(*n* = 12)	(*n* = 5)
ORR, % (80% CI)	7.1 (1.9–17.9)	18.2 (4.9–41.5)	0 (0–17.5)	0 (0–36.9)
Median PFS (80% CI), months	3.7 (1.9–5.6)	5.6 (1.9–7.4)	3.7 (1.9–5.6)	1.6 (NC–NC)
Median time on treatment (80% CI), months^a^	2.2 (1.4–3.2)	3.2 (2.1–5.1)	3.2 (1.4–5.1)	1.3 (0.5–1.4)

Cohort B	(*n* = 13)	(*n* = 7)	(*n* = 5)	(*n* = 1)
CRR, % (80% CI)	7.7 (0.8–26.8)	0 (0–28)	0 (0–36.9)	100 (10–100)
Median radiologic PFS (80% CI), months	3.7 (1.9–NC)	3.7 (NC–NC)	1.9 (1.7–NC)	NC (NC–NC)
Median time on treatment (80% CI), months^a^	1.6 (1.4–3.2)	1.9 (0.8–NC)	1.4 (1.4–1.6)	6 (NC–NC)

a Evaluated as a *post hoc* analysis.

**Table 3.** Individual characteristics of responders who started on ceralasertib 160 mg twice daily.

Cancer	Age, years	*ATM* molecular profile (AF)^a^	ATM IHC, %	Other DDR gene mutations (AF)^a^	Prior systemic therapy	Sites of disease	Best radiologic response	Duration of response, days	Time to onset of OR/CR, days	PSA response	Time on treatment, days
Cohort A	​	​	​	​	​	​	​	​	​	​	​
Breast	55	Biallelic loss: g*ATM*_p.K2756* (53%)	0	g*CHEK2*_splice site 444+1G>A (63%)	Letrozole + palbociclib; fulvestrant + palbociclib; gemcitabine + carboplatin; nab-paclitaxel	Liver	CR	376^b^	54	—	521^b^
Endometrial	65	Biallelic loss: g*ATM*_p.L1162fs*19 (54%) s*ATM*_p.F1774fs*8 (14%)	2	*ARID1A*_p.Q581* (34%) *FANCG*_p.E395fs*5 (43%) *MYC*_amp, *KRAS*_p.G12A (37%)	Paclitaxel + IP cisplatin; liposomal doxorubicin + gemcitabine; carboplatin + gemcitabine	Pelvis, peritoneum, and liver	PR (−56.9%)	57^b^	168	—	168^b^
Cohort B	​	​	​	​	​	​	​	​	​	​	​
mCRPC (Gleason score 9; PSA at entry 82.54 μg/L)	80	*ATM*_p.R3008C (0.3%)	NA	*AR*_amp, *APC*_p. T1556fs*3 (6%), *APC*_del	Leuprolide; bicalutamide; docetaxel; abiraterone; olaparib; CCW702	Prostate, bone, and lung	SD (CTC response)	55	56	Best change from baseline: –42.6% Time to progression: 84 days	183

Abbreviations: AF, allele frequency; CR, composite response; IP, intraperitoneal; NA, not available; OR, objective response.

a Tumor AF for cohort A and ctDNA AF for cohort B.

b Ongoing at data cutoff (December 21, 2022).

**Figure 2.**
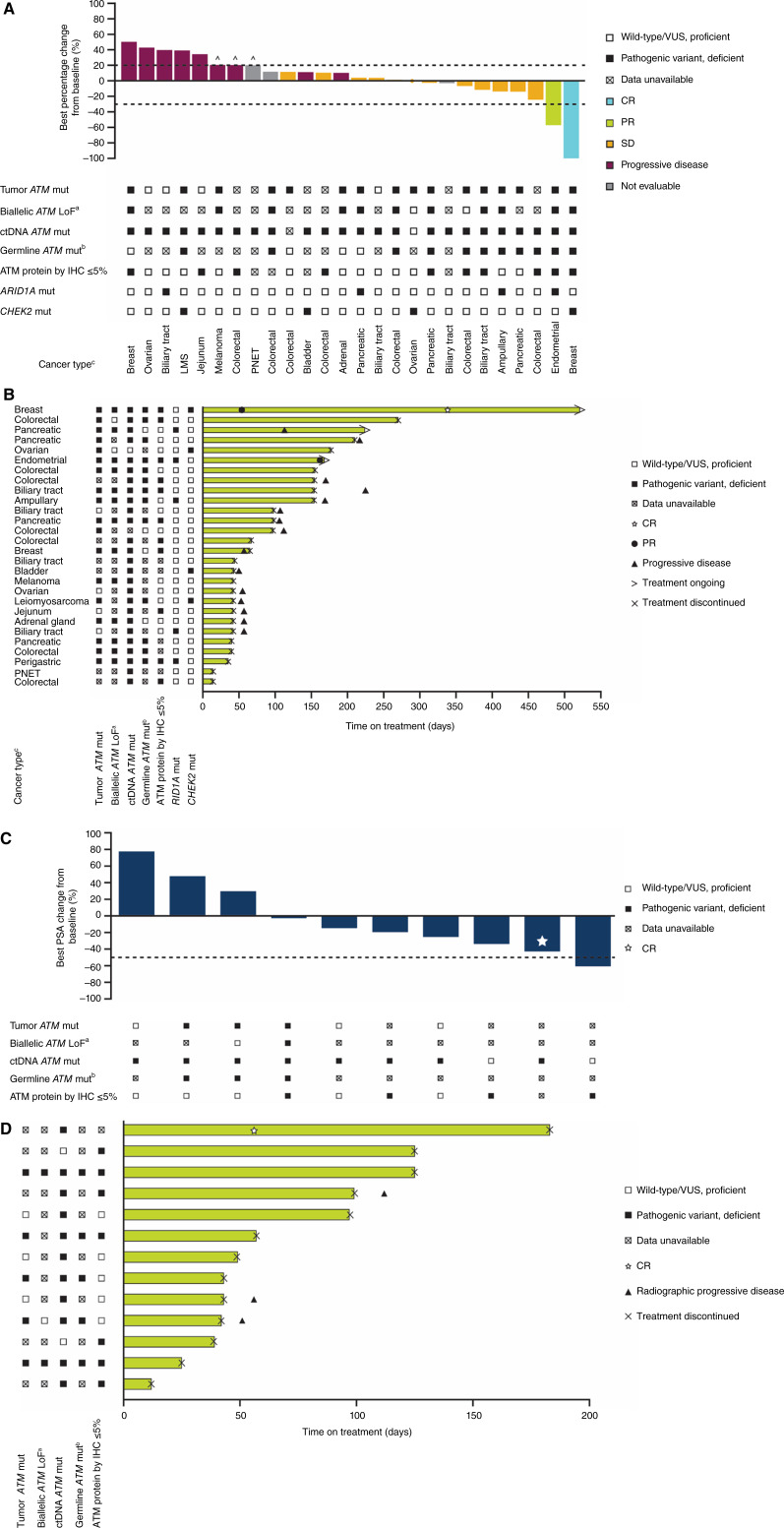
**A,** Best percent change in target lesions (*n* = 26 with postbaseline tumor assessments) and (**B**) time on treatment in all patients with ATM alterations by central testing (*n* = 28) who started on ceralasertib 160 mg twice daily in cohort A (DCO December 21, 2022); (**C**) best percent change in PSA levels (*n* = 10 with post-baseline PSA data) and (**D**) time on treatment in all patients with ATM alterations by central testing (*n* = 13) who started on ceralasertib 160 mg twice daily in cohort B (DCO April 28, 2023). Among patients with ATM alterations by central testing receiving ceralasertib 160 mg twice daily in cohort A, (**A**) two had objective responses (one CR and one PR) and (**B**) treatment was ongoing for three patients at the DCO, including both patients who experienced objective responses. Among patients with ATM alterations by central testing receiving ceralasertib 160 mg twice daily in cohort B, (**C**) one had a composite response, but (**D**) no patients were receiving treatment at the DCO. **A,** Only evaluable or imputed subjects are included in the waterfall plot. Changes near zero are shown with a dot at zero instead of bars. Responses based on RECIST 1.1 (response and progression defined as −30% and +20% change from baseline, respectively). **B,** Time on treatment was evaluated as a *post hoc* analysis. **D,** Time on treatment was evaluated as a *post hoc* analysis. No patients were ongoing treatment at the DCO. ^Patients with imputation where there was a death or evidence of progression. ^a^Based on tumor NGS. ^b^Patients marked as “wild-type/VUS, proficient” for this field had a somatic pathogenic variant. ^c^Derived as a *post hoc* analysis. DCO, data cutoff; LMS, leiomyosarcoma; mut, mutation; PNET, pancreatic neuroendocrine tumor; VUS, variant of uncertain significance.

Among patients with available tumor ATM IHC data in cohort A, the ORR was 18.2% (80% CI, 4.9–41.5) for patients with ≤5% ATM expression (*n* = 2/11) and 0% (80% CI, 0–17.5) for those with >5% ATM expression (*n* = 0/12). Among patients with available biallelic *ATM* LoF status data in cohort A, the ORR was 15.4% (80% CI, 4.2–36) for patients with biallelic LoF (*n* = 2/13) and 0% (80% CI, 0–68.4) for those without (*n* = 0/2). Among patients with available germline *ATM* mutation status data in cohort A, the ORR was 15.4% (80% CI, 4.2–36) for patients with germline mutations (*n* = 2/13) and 0% (80% CI, 0–43.78) for those without (*n* = 0/2). The best percentage change in target lesions for all patients with ATM alterations by central testing in cohort A is shown in Fig. 2A, and the time on treatment in Fig. 2B.

In cohort B, the CRR among patients with ATM alterations by central testing (*n* = 13) was 7.7% (80% CI, 0.8–26.8). The one composite response was recorded due to CTC count conversion from unfavorable to favorable from day 29 to day 84 in a patient with an *ATM* mutation identified by ctDNA NGS but unknown ATM expression by IHC and unknown *ATM* mutation status in his tumor (Table 3). The best objective response in this patient was SD, and the maximum PSA reduction from baseline was −42.6%. Whereas 11 patients with ATM alterations by central testing had measurable disease at baseline, there were no confirmed radiologic responses, and only one unconfirmed response (with a 33% maximum reduction of target lesions). The best percentage change in PSA levels for patients in cohort B is shown in Fig. 2C, and the time on treatment in Fig. 2D. The percentage change in PSA over time for patients in cohort B is shown in Supplementary Fig. S3.

### Progression-free survival

In cohort A, the median PFS was 3.7 months (80% CI, 1.9–5.6) overall (Supplementary Fig. S4A). The median PFS was 5.6 months (80% CI, 1.9–7.4) for patients with ≤5% ATM expression by IHC and 3.7 months (80% CI, 1.9–5.6) for those with >5% ATM expression (Supplementary Fig. S4B).

In cohort B, the median PFS was 3.7 months [80% CI, 1.9–not calculable (NC)] overall (Supplementary Fig. S4C). The median PFS was 3.7 months (80% CI, NC–NC) for patients with ≤5% ATM expression by IHC and 1.9 months (80% CI, 1.7–NC) for those with >5% ATM expression (Supplementary Fig. S4D). Additional PFS data for genetic *ATM* subgroups (including patients with/without biallelic *ATM* LoF and with/without germline *ATM* mutations) in both cohorts are summarized in Supplementary Table S5.

### Safety

The median total treatment duration in patients receiving ceralasertib 160 mg twice daily was 1.8 months (range, 0.5–17.1) in cohort A and 1.9 months (range, 0.4–6) in cohort B. All patients who received ceralasertib 160 mg twice daily had treatment-emergent AEs (Supplementary Table S6). Grade ≥3 AEs occurred in 50% of patients in cohort A and 53.3% of patients in cohort B, and serious AEs occurred in 13.3% and 26.7% of patients, respectively. There were no fatal AEs in either cohort. The most common any-grade AEs across both cohorts were asthenia/fatigue (53.3% in cohort A and 46.7% in cohort B), nausea (43.3% in cohort A and 53.3% in cohort B), and anemia (26.7% in cohort A and 46.7% in cohort B; Table 4).

**Table 4.** Most common AEs (≥15% of patients) among patients who started on ceralasertib 160 mg twice daily in both cohorts.

AE, *n* (%)	Any grade	Grade ≥3
Cohort A (*n* = 30)	​	​
Asthenia/fatigue	16 (53.3)	2 (6.7)
Nausea	13 (43.3)	0
Abdominal pain	9 (30)	0
Thrombocytopenia^a^	8 (26.7)	4 (13.3)
Anemia	8 (26.7)	3 (10)
Decreased appetite	7 (23.3)	1 (3.3)
Constipation	7 (23.3)	0
Diarrhea	6 (20)	1 (3.3)
Dyspnea	5 (16.7)	1 (3.3)
Vomiting	5 (16.7)	1 (3.3)
Stomatitis	5 (16.7)	0
Cohort B (*n* = 15)	​	​
Nausea	8 (53.3)	0
Anemia	7 (46.7)	5 (33.3)
Asthenia/fatigue	7 (46.7)	1 (6.7)
Decreased appetite	3 (20)	0

a Grouped term, includes thrombocytopenia and platelet count decreased preferred terms.

The initial dose of 240 mg twice daily was deemed intolerable and stopped because of high levels of grade 3 to 4 hematologic AEs (neutropenia, febrile neutropenia, thrombocytopenia with or without hemorrhages, and anemia in various combinations, mostly reported during Cycle 1; Supplementary Table S1).

Treatment-related AEs (TRAE) occurred in 70% of patients in cohort A and 86.7% of patients in cohort B receiving ceralasertib 160 mg twice daily (Supplementary Table S6). The most common TRAEs were hematologic (anemia, thrombocytopenia, and neutropenia), gastrointestinal (nausea, decreased appetite, stomatitis, and diarrhea), and asthenia/fatigue. Grade ≥3 TRAEs were reported in 20% and 33.3% of patients, respectively, and serious TRAEs were reported in 6.7% of patients in both cohorts. There were no discontinuations due to serious TRAEs in either cohort.

The most common AEs for patients receiving 160 mg twice daily are summarized by germline *ATM* mutation status in Supplementary Table S7, and the lowest on-study levels of hemoglobin, neutrophil counts, and platelet counts are shown by germline *ATM* mutation status in Supplementary Table S8. Though numbers are small, no apparent safety trends could be identified based on the presence or absence of germline *ATM* mutations.

### Pharmacokinetics

Steady-state ceralasertib concentrations exceeded the IC_90_ value for ∼23 hours per day (Supplementary Fig. S5; ref. 19). Plasma trough concentrations remained relatively constant over the entire dosing cycle.

## Discussion

The PLANETTE trial included two cohorts: cohort A (advanced solid tumors excluding prostate cancer and NSCLC) and cohort B (mCRPC). A dedicated mCRPC cohort was planned due to the anticipated higher prevalence of biallelic *ATM* LoF in prostate cancer (11), and patients with NSCLC were excluded because ceralasertib monotherapy was assessed in *ATM*-altered NSCLC as part of the HUDSON study (NCT03334617; ref. 27).

The PLANETTE study started with 240 mg twice daily on days 1 to 14 of a 28-day cycle ceralasertib regimen that was deemed intolerable due to high-grade hematologic AEs. This contrasts with findings from a phase I study in patients with metastatic solid tumors, which identified ceralasertib 240 mg twice daily on days 1 to 14 of a 28-day cycle as the recommended phase II dose (RP2D) when given with paclitaxel 80 mg/m^2^ on days 1, 8, and 15 (20), highlighting the complexity of optimal dose selection in heterogeneous patient populations based on small dose-escalation cohorts. The PATRIOT study identified 160 mg twice daily on days 1 to 14 of a 28-day cycle as the RP2D for ceralasertib monotherapy, but intermittent dosing at 240 mg was not explored (24). The overall safety profile for intermittent 160 mg dosing in the current study was consistent with that observed in PATRIOT and prior clinical evaluations of ceralasertib (6, 20–23), with few serious TRAEs and no serious TRAEs leading to treatment discontinuation. Consistent with clinical studies of other ATR inhibitors as monotherapy, hematologic AEs such as anemia were common in PLANETTE, highlighting them as a potential drug-class effect (2).

Responses to ceralasertib monotherapy in PLANETTE were limited despite reaching target plasma levels, and cohort B was terminated early following a final analysis of cohort A and an *ad hoc* analysis of cohort B. Given the need for measurable disease and/or an unfavorable CTC count, it is possible that cohort B selected for patients with more advanced disease (i.e., patients with metastatic lesions in areas other than bone, including visceral metastatic lesions). The highest ORR and longest median PFS in cohort A were both observed in patients with ATM protein–deficient tumors (≤5% ATM expression by IHC), and no responses were observed in patients with >5% ATM expression in their tumors. Responses were also observed in patients with *ATM* mutations identified by tumor and ctDNA NGS, but further analyses in genetic *ATM* subgroups (including patients with biallelic *ATM* LoF and germline *ATM* mutations) were limited by small patient numbers and lacked statistical power. Of note, ATM alterations in some patients were identified in ctDNA but were either not detected (*n* = 3 in cohort A and *n* = 3 in cohort B) or unknown (*n* = 3 in cohort A and *n* = 1 in cohort B) in tumor tissue samples, raising the possibility that alterations identified in ctDNA, but not in tumor tissue samples, could be attributed to clonal hematopoiesis, which was not assessed in this study. Recent results suggest that approximately 9% of *ATM* mutations detected in liquid biopsies may be attributable to CHIP (28), but this number may be much higher (62%) in prostate cancer (29). Thus, future studies guided by *ATM* mutations should incorporate appropriate controls to exclude patients whose mutations originate from CHIP.

Interpretations of response data for ceralasertib monotherapy in PLANETTE may be confounded by the presence of additional DDR gene alterations in tumors. In cohort A, the responders with breast cancer and endometrial cancer had concurrent pathogenic variants of *CHEK2* (encoding CHK2, a downstream target of ATM) and *ARID1A*, respectively, in their tumors. Preclinical studies have identified markers associated with replication stress and other DDR alterations, such as mutations in *ARID1A* and members of the MRE11–RAD50–NBS1 (MRN) complex (required for ATM activation), that may sensitize tumors to ATR inhibition (19, 30–33). A recent clinical study also demonstrated ceralasertib activity in patients with ARID1A-deficient tumors, including two CRs in ARID1A-deficient endometrial cancers with sustained and long-term clinical benefit (34). Similarly, the ATARI study provided evidence for ceralasertib monotherapy activity in gynecologic cancers with deleterious *ARID1A* mutations (35). Durable clinical responses were also observed in several patients with tumors harboring DDR gene defects (including *ARID1A* and *MRE11* mutations) receiving ceralasertib monotherapy in the PATRIOT study (24).

Results have been reported from clinical studies of other ATR inhibitors such as elimusertib (BAY1895344; refs. 14, 25) and camonsertib (RP-3500; refs. 15, 26) since PLANETTE was initiated. These studies highlighted monotherapy activity in ATM-selected populations and have demonstrated similarly modest outcomes to PLANETTE in terms of limited ORRs but durable clinical benefit in a subset of patients.

In a dose-expansion cohort of a phase Ib elimusertib trial that selected for ATM protein loss by IHC (with a ≤10% positivity cutoff) across tumor types, the ORR was 8.8% (*n* = 3/34) and the clinical benefit rate (CBR; defined as best response of CR + PR + SD lasting ≥120 days) was 44.1% (*n* = 15/34; ref. 25). Interestingly, several of the responders had tumors harboring other DDR mutations, including *ARID1A* and *FANCA*. However, ATM protein loss alone did not predict clinical benefit or longer PFS with elimusertib. Similarly, ATM protein deficiency in cohort A of PLANETTE did not predict prolonged PFS or time on treatment, though two objective responses were observed.

In a phase I dose-escalation study of camonsertib, the response rate (assessed by RECIST 1.1, PSA, or CA-125) was 15.2% (*n* = 7/46: *n* = 5 RECIST 1.1 responses and *n* = 3 PSA molecular responses; one patient experienced both a RECIST 1.1 and a PSA response) and the CBR was 47.8% (*n* = 22/46) in the subset of efficacy-evaluable patients with ATM alterations (defined as having an *ATM* mutations and/or ATM protein expression <10% by IHC; refs. 15, 26). All seven responders had tumors with biallelic *ATM* LoF by central testing (*n* = 5 germline and *n* = 2 somatic; ref. 15). In PLANETTE, both responders in cohort A also had biallelic *ATM* LoF, and no responses were observed in patients without biallelic *ATM* LoF.

Together, these studies indicate that *ATM* mutations or ATM protein loss alone may not be sufficient to predict clinical benefit with ATR inhibitor monotherapy and underscore the need for an optimized patient selection strategy.

Ceralasertib has also been assessed as a component of combination therapies (21–23, 27). In one arm of the OLAPCO study, ceralasertib was combined with olaparib based on preclinical evidence supporting the combination in BRCA-deficient cancers (21, 36, 37). The CBR was 40% among ATM-altered patients (*n* = 2/5; CR and SD, both lasting ≥26 months), and the most durable responses occurred in patients with germline *ATM* mutations and evidence of tumoral biallelic *ATM* LoF. However, it is difficult to distinguish the individual contributions of olaparib and ceralasertib (21). In the phase II HUDSON study in patients with advanced NSCLC, ceralasertib in combination with the immune checkpoint inhibitor durvalumab showed numerically higher (but not statistically significant) ORR, median PFS, and median OS in *ATM*-altered patients compared with those without ATM alterations (27). Similarly, the ATR inhibitor ART0380 in combination with irinotecan demonstrated objective responses in 22.8% of 57 patients with advanced cancers, with an ORR of 45% in patients with ATM protein loss (38). Another ATR inhibitor, M6620 (berzosertib), induced a PR in one of 21 evaluable patients when combined with carboplatin (in a patient with advanced, germline *BRCA1*-mutated ovarian cancer; a nonpathogenic *ATM* mutation was also identified in a tumor sample from this patient; ref. 39). Thus, although *ATM* mutations and/or protein loss may help to identify responders to ATR inhibitor monotherapy, alternative biomarkers and combination approaches with ceralasertib may represent more promising strategies to improve on the limited clinical activity observed to date.

### Conclusions

Responses to ceralasertib were limited in PLANETTE, which is consistent with previous observations for other ATR inhibitor monotherapies, but the 160 mg twice daily regimen demonstrated manageable safety consistent with prior clinical evaluations. Optimizing biomarker-based patient selection beyond ATM deficiency represents a key aspect of future ATR inhibitor development.

## Supplementary Material

Supplementary Table 1 Adverse events observed in ≥2 patients who started on ceralasertib 240 mg BID in Cohort

Supplementary Table 2 Baseline disease characteristics for patients with ATM alterations by central testing who started on ceralasertib 160 mg BID in Cohort B

Supplementary Table 3 Representativeness of study participants

Supplementary Table 4 Prevalence of ATM alterations identified in tumors (NGS or IHC) versus ctDNA only (NGS) among patients with ATM alterations by central testing who started on ceralasertib 160 mg BID

Supplementary Table 5 Ceralasertib 160 mg BID efficacy and time on treatment in genetic ATM subgroups determined by central testing

Supplementary Table 6 Safety summary for all patients who started on ceralasertib 160 mg BID

Supplementary Table 7 Most common AEs occurring at any grade in ≥15% of patients who started on ceralasertib 160 mg BID in each cohort by germline ATM mutation status

Supplementary Table 8 Lowest hemoglobin levels, neutrophil counts, and platelet counts in patients who started on ceralasertib 160 mg BID by germline ATM mutation status

Supplementary Figure 1 CONSORT diagram for patients who started on ceralasertib 240 mg BID

Supplementary Figure 2 MRI scan showing target lesion reduction in the responder with endometrial cancer receiving ceralasertib 160 mg BID in Cohort A

Supplementary Figure 3 Percentage change in PSA over time for patients with ATM alterations by central testing who started on ceralasertib 160 mg BID in Cohort B

Supplementary Figure 4 Progression-free survival for (a) patients who started on ceralasertib 160 mg BID in Cohort A, (b) patients with ATM protein expression ≤5% versus >5% versus unknown by IHC who started on ceralasertib 160 mg BID in Cohort A, (c) patients who started on ceralasertib 160 mg BID in Cohort B, and (d) patients with ATM protein expression ≤5% versus ATM >5% versus unknown by IHC who started on ceralasertib 160 mg BID in Cohort B

Supplementary Figure 5 Geometric mean plasma concentrations of ceralasertib on Day 8 and Day 14 of Cycle 1 (blue) and Cycle 2 (orange) following receipt of ceralasertib 160 mg administered BID on days 1–14 of a 28-day cycle

## Data Availability

Data underlying the findings described in this manuscript may be obtained in accordance with AstraZeneca’s data sharing policy described at https://astrazenecagrouptrials.pharmacm.com/ST/Submission/Disclosure. Data for studies not listed on Vivli could be requested through Vivli at https://vivli.org/members/enquiries-about-studies-not-listed-on-the-vivli-platform/. AstraZeneca Vivli member page is also available outlining further details: https://vivli.org/ourmember/astrazeneca/.
