# Supplementary material for: Ceralasertib Monotherapy in Patients with ATM-Altered Advanced Solid Tumors or Metastatic Castration-Resistant Prostate Cancer: Data from the Phase IIa PLANETTE Study
Source: Cancer Res Commun. 2026 Jul 2;6(7):1546–56. doi: 10.1158/2767-9764.CRC-26-0184 (PMC13324620; doi:10.1158/2767-9764.CRC-26-0184)
Supplement: Supplementary Table 1 — Adverse events observed in ≥2 patients who started on ceralasertib 240 mg BID in Cohort [file crc-26-0184_supplementary_table_1_suppst1.pdf]

Supplementary Table 1. Adverse events observed in ≥2 patients who started on ceralasertib 240 mg BID in Cohort A

AE, n (%)	Any grade	Grade ≥3
**Cohort A (n = 8)**		
Any	8 (100)	6 (75.0)
Anemia	6 (75.0)	3 (37.5)[a]
Dyspnea	4 (50.0)	0
Fatigue	4 (50.0)	0
Nausea	4 (50.0)	0
White blood cell count decreased	4 (50.0)	2 (25.0)[b]
Neutrophil count decreased	3 (37.5)	3 (37.5)[c]
Platelet count decreased	3 (37.5)	2 (25.0)[d]
Vomiting	3 (37.5)	0
Thrombocytopenia	2 (25.0)	2 (25.0)[e]
Hypoxia	2 (25.0)	1 (12.5)[f]
Rash maculopapular	2 (25.0)	1 (12.5)[g]
Abdominal pain	2 (25.0)	0
AST increased	2 (25.0)	0
Constipation	2 (25.0)	0
Cough	2 (25.0)	0
Decreased appetite	2 (25.0)	0
Hypoalbuminemia	2 (25.0)	0
Hyponatremia	2 (25.0)	0
Oedema peripheral	2 (25.0)	0

[a]All grade 3 events; [b]Both grade 3 events; [c]All grade 3 events; [d]Both grade 4 events; [e]One grade 3 event and one grade 4 event; [f]Grade 3; [g]Grade 3.

AST, aspartate aminotransferase; BID, twice daily.
