# Supplementary material for: Ceralasertib Monotherapy in Patients with ATM-Altered Advanced Solid Tumors or Metastatic Castration-Resistant Prostate Cancer: Data from the Phase IIa PLANETTE Study
Source: Cancer Res Commun. 2026 Jul 2;6(7):1546–56. doi: 10.1158/2767-9764.CRC-26-0184 (PMC13324620; doi:10.1158/2767-9764.CRC-26-0184)
Supplement: Supplementary Table 2 — Baseline disease characteristics for patients with ATM alterations by central testing who started on ceralasertib 160 mg BID in Cohort B [file crc-26-0184_supplementary_table_2_suppst2.pdf]

Supplementary Table 2. Baseline disease characteristics for patients with ATM alterations by central testing who started on ceralasertib 160 mg BID in Cohort B

Parameter	Cohort B (n = 13)
PSA, median (IQR), ng/mL	70.0 (14.1–247.0)
Metastatic site, n (%)	
Bone or muscle but not visceral	8 (61.5)
Visceral except liver	4 (30.8)
Liver	1 (7.7)
Gleason score at initial diagnosis, n (%)	
≤6	1 (7.7)
7	3 (23.1)
≥8	8 (61.5)
Unknown	1 (7.7)
CTC count, n (%)	
Favorable (<5 cells per 7.5 mL blood)	3 (23.1)
Unfavorable (≥5 cells per 7.5 mL blood)	6 (46.2)
Unknown	4 (30.8)
Time since initial diagnosis, median (IQR), years	5.12 (3.0–11.7)
Time since CRPC diagnosis, median (IQR), years	1.85 (0.5–3.7)
Prior therapies, n (%)	
Taxanes	11 (84.6)
PARP inhibitors	6 (46.2)
Systemic radioisotope therapy	2 (15.4)

ATM, ataxia-telangiectasia mutated; BID, twice daily; CTC, circulating tumor cell; CRPC, castration-resistant prostate cancer; IQR, interquartile range; PARP, poly-ADP ribose polymerase; PSA, prostate-specific antigen.
