# Supplementary material for: Ceralasertib Monotherapy in Patients with ATM-Altered Advanced Solid Tumors or Metastatic Castration-Resistant Prostate Cancer: Data from the Phase IIa PLANETTE Study
Source: Cancer Res Commun. 2026 Jul 2;6(7):1546–56. doi: 10.1158/2767-9764.CRC-26-0184 (PMC13324620; doi:10.1158/2767-9764.CRC-26-0184)
Supplement: Supplementary Table 3 — Representativeness of study participants [file crc-26-0184_supplementary_table_3_suppst3.pdf]

Supplementary Table 3. Representativeness of study participants

**Cancer type(s) / subtype(s) / stage(s) / condition**	Advanced solid tumors excluding non-small-cell lung cancer or prostate cancer (n = 28 in Cohort A) and mCRPC (n = 13 in Cohort B) with progression on prior treatment, no standard treatment options, and an *ATM* mutation identified by a pre-existing local tumor or blood test
**Considerations related to:**	
**Sex**	• All cancers: Cancer rates are higher in males versus females (478.1 versus 422.4 per 100,000 in the US in 2022) (1).<br>• mCRPC: Prostate cancer is the most frequently diagnosed cancer among males in 112 countries, including the US (2, 3). In 2022, the prostate cancer incidence rate was 124.5 per 100,000 in the US (1) and the age-standardized global rate was 29.4% (4).
**Age**	• All cancers: The median age at cancer diagnosis is 67.0 years: 68.0 for males and 66.0 for females (1).<br>• mCRPC: The median age at diagnosis of prostate cancer is 68.0 years (1). Per Medicare data, the mean age of patients with mCRPC in the US is 76.1 years (5); however, Medicare patients may not be considered representative of all US patients with prostate cancer.
**Race/ethnicity**	• All cancers: Per US data from 2022, non-Hispanic White people have the highest cancer incidence rate (473.3 per 100,000), followed by non-Hispanic Black people (455.1 per 100,000), non-Hispanic American Indians/Alaska Natives (411.2 per 100,000), Hispanic people (any race; 359.0 per 100,000), and non-Hispanic Asian people/Pacific Islanders (314.9 per 100,000) (1).<br>• mCRPC: Per US data from 2022, non-Hispanic Black people have the highest prostate cancer incidence rate (198.1 per 100,000), followed by non-Hispanic White people (122.2 per 100,000), Hispanic people (any race; 91.8 per 100,000), non-Hispanic American Indians/Alaska Natives (89.1 per 100,000), and non-Hispanic Asian people/Pacific Islanders (68.6 per 100,000) (1).
**Geography**	• All cancers: In 2025, an estimated 2,041,910 new cancer cases will be diagnosed in the US, resulting in an estimated 618,120 cancer-related deaths (6).<br>• mCRPC: North America and Western Europe were among the regions with the highest age-standardized rates of prostate cancer per GLOBOCAN 2022 data (73.5 and 65.7 per 100,000, respectively) (7). In 2025, an estimated 313,780 new cases of prostate cancer will be diagnosed in the US (15.4% of all new cancer cases) (8).
**Other considerations**	• All cancers: None.

	• mCRPC: Approximately 75% of patients have localized prostate cancer at diagnosis (9) and ~10–20% of patients diagnosed with prostate cancer develop mCRPC within 5 years following initial therapy (10). Additional risk factors for prostate cancer include family history, genetic predisposition, and diet (meat intake, trans fatty acid intake, and dietary inflammatory index); smoking, diet, physical activity, and other medications may also contribute (11).
**Overall representativeness of the study**	It should be noted that this study was small (N = 41 patients overall; 28 in Cohort A and 13 in Cohort B), which impacts the representativeness of the patients enrolled. Individual disease groups in Cohort A included ≤7 patients each and data for Cohort A are pooled across multiple indications, which limits comparisons with the wider disease populations. The enrolled population in Cohort B had a median age of 72.0 years; this is aligned with the SEER-reported value of 68.0 years at prostate cancer diagnosis (1) when accounting for the expected delay between initial prostate cancer diagnosis and development of metastases and castration resistance (10). In total, 53.8% of patients in Cohort B had no available race data; those with available race data were all White, which limits the applicability of the results to patients with mCRPC of other racial and ethnic groups.

References

1. National Cancer Institute. Surveillance, Epidemiology, and End Results Program. Available at: https://seer.cancer.gov/statistics-network/explorer/application.html. Accessed June 10, 2025.
2. James ND, Tannock I, N'Dow J, Feng F, Gillessen S, Ali SA, et al. The *Lancet* Comission on prostate cancer: planning for the surge in cases. Lancet 2024;403:1683–722.
3. National Cancer Institute. Surveillance, Epidemiology, and End Results Program. Cancer Stat Facts: Common Cancer Sites. Available at: https://seer.cancer.gov/statfacts/html/common.html. Accessed June 10, 2025.
4. Bray F, Laversanne M, Sung H, Ferlay J, Siegel RL, Soerjomataram I, et al. Global cancer statistics 2022: GLOBOCAN estimates of incidence and mortality worldwide for 36 cancers in 185 countries. CA Cancer J Clin 2024;74:229–63.
5. Freedland SJ, Davis M, Epstein AJ, Arondekar B, Ivanova JI. Real-world treatment patterns and overall survival among men with Metastatic Castration-Resistant Prostate Cancer (mCRPC) in the US Medicare population. Prostate Cancer Prostatic Dis 2024;27:327–33.
6. National Cancer Institute. Surveillance, Epidemiology, and End Results Program. Cancer Stat Facts: Cancer of Any Site. Available at: https://seer.cancer.gov/statfacts/html/all.html. Accessed June 25, 2025.
7. Schafer EJ, Laversanne M, Sung H, Soerjomataram I, Briganti A, Dahut W. Recent Patterns and Trends in Global Prostate Cancer Incidence and Mortality: An Update. Eur Urol 2025;87:302–13.
8. National Cancer Institute. Surveillance, Epidemiology, and End Results Program. Cancer Stat Facts: Prostate Cancer. Available at: https://seer.cancer.gov/statfacts/html/prost.html. Accessed June 10, 2025.
9. Raychaudhuri R, Lin DW, Montgomery RB. Prostate Cancer: A Review. JAMA 2025;333:1433–46.

10. Merseburger AS, Bellmunt J, Jenkins C, Parker C, Fitzpatrick JM. Perspectives on treatment of metastatic castration-resistant prostate cancer. Oncologist 2013;18:558–67.
11. Bergengren O, Pekala KR, Matsoukas K, Fainberg J, Mungovan SF, Bratt O, et al. 2022 Update on Prostate Cancer Epidemiology and Risk Factors – A Systematic Review. Eur Urol 2023;84:191–206.

ATM, ataxia-telangiectasia mutated; GLOBOCAN, Global Cancer Observatory; mCRPC, metastatic castration-resistant prostate cancer; SEER, Surveillance, Epidemiology, and End Results; US, United States.
