# Supplementary material for: Ceralasertib Monotherapy in Patients with ATM-Altered Advanced Solid Tumors or Metastatic Castration-Resistant Prostate Cancer: Data from the Phase IIa PLANETTE Study
Source: Cancer Res Commun. 2026 Jul 2;6(7):1546–56. doi: 10.1158/2767-9764.CRC-26-0184 (PMC13324620; doi:10.1158/2767-9764.CRC-26-0184)
Supplement: Supplementary Table 4 — Prevalence of ATM alterations identified in tumors (NGS or IHC) versus ctDNA only (NGS) among patients with ATM alterations by central testing who started on ceralasertib 160 mg BID [file crc-26-0184_supplementary_table_4_suppst4.pdf]

Supplementary Table 4. Prevalence of ATM alterations identified in tumors (NGS or IHC) versus ctDNA only (NGS) among patients with ATM alterations by central testing who started on ceralasertib 160 mg BID

**Parameter**	**Cohort A (n = 28)**	**Cohort B (n = 13)**
ATM mutation in tumor by NGS and/or ATM protein loss by IHC (regardless of ctDNA *ATM* status)	n = 22	n = 9
Positive by ctDNA NGS, n (%)	20 (90.9)	7 (77.8)
Negative by ctDNA NGS, n (%)	1 (4.5)	2 (22.2)
Unknown by ctDNA NGS, n (%)	1 (4.5)	0
ATM mutation in ctDNA by NGS only (negative/unknown by tumor NGS and/or IHC)	n = 6	n = 4
Negative by both tumor NGS and IHC	3 (50.0)	3 (75.0)
Unknown by both tumor NGS and IHC	3 (50.0)	1 (25.0)

ATM, ataxia-telangiectasia mutated; BID, twice daily; ctDNA, circulating tumor DNA; IHC, immunohistochemistry; NGS, next-generation sequencing.
