# Supplementary material for: Ceralasertib Monotherapy in Patients with ATM-Altered Advanced Solid Tumors or Metastatic Castration-Resistant Prostate Cancer: Data from the Phase IIa PLANETTE Study
Source: Cancer Res Commun. 2026 Jul 2;6(7):1546–56. doi: 10.1158/2767-9764.CRC-26-0184 (PMC13324620; doi:10.1158/2767-9764.CRC-26-0184)
Supplement: Supplementary Table 5 — Ceralasertib 160 mg BID efficacy and time on treatment in genetic ATM subgroups determined by central testing [file crc-26-0184_supplementary_table_5_suppst5.pdf]

Supplementary Table 5. Ceralasertib 160 mg BID efficacy and time on treatment in genetic *ATM* subgroups determined by central testing

***ATM* status and outcome**	**Cohort A (n = 28)**	**Cohort B (n = 13)**
Biallelic *ATM* loss-of-function		
Yes, n	13	2
ORR/CRR, % (80% CI)[a]	15.4 (4.2–36.0)	0 (0–68.4)
Median PFS (80% CI), months[b]	4.7 (3.5–7.4)	NC (NC–NC)
Median time on treatment (80% CI), months	3.2 (1.4–5.1)	2.5 (0.8–NC)
No, n	2	1
ORR/CRR, % (80% CI)[a]	0 (0–68.4)	0 (0–90.0)
Median PFS (80% CI), months[b]	NC (NC–NC)	1.7 (NC–NC)
Median time on treatment (80% CI), months	7.3 (5.8–NC)	1.4 (NC–NC)
Unknown, n	13	10
ORR/CRR, % (80% CI)[a]	0 (0–16.2)	10.0 (1.0–33.7)
Median PFS (80% CI), months[b]	1.9 (1.8–3.7)	NC (1.9–NC)
Median time on treatment (80% CI), months	1.4 (1.4–2.2)	1.7 (1.4–3.2)
Germline *ATM* mutation		
Yes, n	13	5
ORR/CRR, % (80% CI)[a]	15.4 (4.2–36.0)	0 (0–36.9)
Median PFS (80% CI), months[b]	7.4 (5.6–7.4)	NC (1.7–NC)
Median time on treatment (80% CI), months	5.1 (1.4–5.1)	1.4 (0.8–1.9)
No, n	4	0
ORR/CRR, % (80% CI)[a]	0 (0–43.8)	–
Median PFS (80% CI), months[b]	2.8 (1.9–NC)	–
Median time on treatment (80% CI), months	2.7 (1.4–NC)	–
Unknown, n	11	8
ORR/CRR, % (80% CI)[a]	0 (0–18.9)	12.5 (1.3–40.6)
Median PFS (80% CI), months[b]	1.9 (1.8–3.5)	NC (1.9–NC)
Median time on treatment (80% CI), months	1.4 (1.4–1.4)	2.4 (1.3–3.3)
ATM mutation in tumor by NGS and/or ATM protein loss by IHC (regardless of ctDNA *ATM* status)		
Yes, n	22	9
ORR/CRR, % (80% CI)[a]	9.1 (2.4–22.4)	0 (0–22.6)
Median PFS (80% CI), months[b]	5.6 (3.7–7.4)	3.7 (1.7–NC)
Median time on treatment (80% CI), months	3.2 (1.4–5.1)	1.4 (1.3–3.3)
ATM mutation in ctDNA by NGS only (negative/unknown by tumor NGS and/or IHC)		
Yes, n	6	4
ORR/CRR, % (80% CI)[a]	0 (0–31.9)	25.0 (2.6–68.0)
Median PFS (80% CI), months[b]	1.8 (1.6–NC)	NC (1.9–NC)
Median time on treatment (80% CI), months	1.4 (1.4–1.4)	2.4 (1.4–NC)

[a]ORRs shown for Cohort A and CRRs shown for Cohort B.

[b]Radiological PFS shown for Cohort B.

ATM, ataxia-telangiectasia mutated; BID, twice daily; CI, confidence interval; CRR, composite response rate; ctDNA, circulating tumor DNA; IHC, immunohistochemistry; NC, not calculable; NGS, next-generation sequencing; ORR, objective response rate; PFS, progression-free survival.
