# Supplementary material for: Ceralasertib Monotherapy in Patients with ATM-Altered Advanced Solid Tumors or Metastatic Castration-Resistant Prostate Cancer: Data from the Phase IIa PLANETTE Study
Source: Cancer Res Commun. 2026 Jul 2;6(7):1546–56. doi: 10.1158/2767-9764.CRC-26-0184 (PMC13324620; doi:10.1158/2767-9764.CRC-26-0184)
Supplement: Supplementary Table 6 — Safety summary for all patients who started on ceralasertib 160 mg BID [file crc-26-0184_supplementary_table_6_suppst6.pdf]

Supplementary Table 6. Safety summary for all patients who started on ceralasertib 160 mg BID

Parameter, n (%)	Cohort A (n = 30)	Cohort B (n = 15)
Any AEs	30 (100)	15 (100)
Grade ≥3 AEs	15 (50.0)	8 (53.3)
Serious AEs	4 (13.3)	4 (26.7)
AEs leading to dose interruption	7 (23.3)	3 (20.0)
AEs leading to dose reduction	4 (13.3)	3 (20.0)
AEs leading to treatment discontinuation	1 (3.3)[a]	1 (6.7)
AEs leading to death	0	0
Any TRAEs	21 (70.0)	13 (86.7)
Grade ≥3 TRAEs	6 (20.0)	5 (33.3)
Serious TRAEs	2 (6.7)	1 (6.7)
TRAEs leading to treatment discontinuation	0	1 (6.7)
TRAEs leading to death	0	0

[a]Discontinuation due to decreased appetite classified as a serious adverse event.

AE, adverse event; BID, twice daily; TRAE, treatment-related adverse event.
