# Supplementary material for: Ceralasertib Monotherapy in Patients with ATM-Altered Advanced Solid Tumors or Metastatic Castration-Resistant Prostate Cancer: Data from the Phase IIa PLANETTE Study
Source: Cancer Res Commun. 2026 Jul 2;6(7):1546–56. doi: 10.1158/2767-9764.CRC-26-0184 (PMC13324620; doi:10.1158/2767-9764.CRC-26-0184)
Supplement: Supplementary Table 7 — Most common AEs occurring at any grade in ≥15% of patients who started on ceralasertib 160 mg BID in each cohort by germline ATM mutation status [file crc-26-0184_supplementary_table_7_suppst7.pdf]

Supplementary Table 7. Most common AEs occurring at any grade in ≥15% of patients who started on ceralasertib 160 mg BID in each cohort by germline *ATM* mutation status

AE, n (%)	Germline *ATM* mutation		No germline *ATM* mutation		Unknown germline *ATM* mutation status		All	
	**Any grade**	**Grade ≥3**	**Any grade**	**Grade ≥3**	**Any grade**	**Grade ≥3**	**Any grade**	**Grade ≥3**
**Cohort A**	**(n = 13)**		**(n = 4)**		**(n = 13)**		**(n = 30)**	
Asthenia/fatigue	10 (76.9)	2 (15.4)	1 (25.0)	0	5 (38.5)	0	16 (53.3)	2 (6.7)
Nausea	6 (46.2)	0	2 (50.0)	0	5 (38.5)	0	13 (43.3)	0
Abdominal pain	5 (38.5)	0	2 (50.0)	0	2 (15.4)	0	9 (30.0)	0
Thrombocytopenia[a]	2 (15.4)	1 (7.7)	1 (25.0)	0	5 (38.5)	3 (23.1)	8 (26.7)	4 (13.3)
Anemia	5 (38.5)	2 (15.4)	1 (25.0)	0	2 (15.4)	1 (7.7)	8 (26.7)	3 (10.0)
Decreased appetite	2 (15.4)	1 (7.7)	1 (25.0)	0	4 (30.8)	0	7 (23.3)	1 (3.3)
Constipation	3 (23.1)	0	1 (25.0)	0	3 (23.1)	0	7 (23.3)	0
Diarrhea	5 (38.5)	1 (7.7)	0	0	1 (7.7)	0	6 (20.0)	1 (3.3)
Vomiting	2 (15.4)	0	1 (25.0)	1 (25.0)	2 (15.4)	0	5 (16.7)	1 (3.3)
Dyspnea	0	0	1 (25.0)	0	4 (30.8)	1 (7.7)	5 (16.7)	1 (3.3)
Stomatitis	0	0	2 (50.0)	0	3 (23.1)	0	5 (16.7)	0
**Cohort B**	**(n = 5)**		**(n = 0)**		**(n = 10)**		**(n = 15)**	
Nausea	3 (60.0)	0	–	–	5 (50.0)	0	8 (53.3)	0
Anemia	1 (20.0)	1 (20.0)	–	–	6 (60.0)	4 (40.0)	7 (46.7)	5 (33.3)
Asthenia/fatigue	2 (40.0)	0	–	–	5 (50.0)	1 (10.0)	7 (46.7)	1 (6.7)
Decreased appetite	0	0	–	–	3 (30.0)	0	3 (20.0)	0

[a]Grouped term, includes thrombocytopenia and platelet count decreased preferred terms.

AE, adverse event; ATM, ataxia-telangiectasia mutated; BID, twice daily.
