# Supplementary material for: Ceralasertib Monotherapy in Patients with ATM-Altered Advanced Solid Tumors or Metastatic Castration-Resistant Prostate Cancer: Data from the Phase IIa PLANETTE Study
Source: Cancer Res Commun. 2026 Jul 2;6(7):1546–56. doi: 10.1158/2767-9764.CRC-26-0184 (PMC13324620; doi:10.1158/2767-9764.CRC-26-0184)
Supplement: Supplementary Table 8 — Lowest hemoglobin levels, neutrophil counts, and platelet counts in patients who started on ceralasertib 160 mg BID by germline ATM mutation status [file crc-26-0184_supplementary_table_8_suppst8.pdf]

Supplementary Table 8. Lowest hemoglobin levels, neutrophil counts, and platelet counts in patients who started on ceralasertib 160 mg BID by germline *ATM* mutation status

	Cohort A (n = 30)				Cohort B (n = 15)		
**Parameter**	**Germline *ATM* mutation (n = 13)**	**No germline *ATM* mutation (n = 4)**	**Unknown germline *ATM* mutation status (n = 13)**	**All (n = 30)**	**Germline *ATM* mutation (n = 5)**	**Unknown germline *ATM* mutation status (n = 10)**	**All (n = 15)**
Hb							
Hb at baseline, g/L							
Mean (SD)	114.1 (15.4)	123.5 (20.4)	122.8 (16.5)	119.1 (16.5)	115.6 (15.8)	109.8 (15.9)	111.7 (15.5)
Median (IQR)	116.5 (99.5–124.5)	118.5 (107.5–139.5)	122.0 (115.5–134.0)	119.5 (107.5–129.0)	117.0 (116.0–122.0)	113.5 (93.0–123.0)	117.0 (93.0–123.0)
Patients with lowest Hb value, n (%)							
<LLN	12 (29.3)	4 (100)	11 (84.6)	27 (90.0)	5 (100)	9 (90.0)	14 (93.3)
100 g/L to LLN	4 (30.8)	1 (25.0)	5 (38.5)	10 (33.3)	3 (60.0)	3 (30.0)	6 (40.0)
80 to 100 g/L	7 (53.8)	3 (75.0)	4 (30.8)	14 (46.7)	1 (20.0)	2 (20.0)	3 (20.0)
<80 g/L	1 (7.7)	0	2 (15.4)	3 (10.0)	1 (20.0)	4 (40.0)	5 (33.3)
Neutrophil counts							
Neutrophil count at baseline, 10^9/L							
Mean (SD)	5.5 (1.6)	5.0 (1.1)	6.6 (5.3)	5.9 (3.6)	3.6 (1.5)	4.8 (1.4)	4.4 (1.5)
Median (IQR)	5.1 (5.0–6.4)	5.4 (4.3–5.7)	4.8 (4.0–7.5)	5.0 (4.5–5.8)	2.7 (2.5–4.7)	4.7 (4.1–5.8)	4.6 (2.7–5.8)
Patients with lowest neutrophil count, n (%)							
<LLN	3 (23.1)	1 (25.0)	0	4 (13.3)	2 (40.0)	2 (20.0)	4 (26.7)
1.5 x 10^9/L to LLN	3 (23.1)	1 (25.0)	0	4 (13.3)	1 (20.0)	1 (10.0)	2 (13.3)
1 to 1.5 x 10^9/L	0	0	0	0	1 (20.0)	1 (10.0)	2 (13.3)
0.5 to 1 x 10^9/L	0	0	0	0	0	0	0
<0.5 x 10^9/L	0	0	0	0	0	0	0
Platelet counts							
Platelet count at baseline, 10^9/L							
Mean (SD)	260.9 (91.9)	370.0 (35.4)	282.9 (85.1)	280.8 (87.9)	185.4 (46.0)	274.6 (54.5)	244.9 (66.4)
Median (IQR)	241.5 (211.0–293.0)	370.0 (345.0–395.0)	276.0 (217.0–337.0)	276.0 (217.0–345.0)	184.0 (148.0–222.0)	273.0 (230.0–321.0)	240.0 (192.0–302.0)
Patients with lowest platelet count, n (%)							
<LLN	3 (23.1)	0	5 (38.5)	8 (26.7)	4 (80.0)	4 (40.0)	8 (53.3)
75 x 10^9/L to LLN	2 (15.4)	0	3 (23.1)	5 (16.7)	4 (80.0)	4 (40.0)	8 (53.3)
50 to 75 x 10^9/L	0	0	2 (15.4)	2 (6.7)	0	0	0
25 to 50 x 10^9/L	1 (7.7)	0	0	1 (3.3)	0	0	0
<25 x 10^9/L	0	0	0	0	0	0	0

ATM, ataxia-telangiectasia mutated; BID, twice daily; Hb, hemoglobin; IQR, interquartile range; LLN, lower limit of normal; SD, standard deviation.
