# Supplementary material for: Ceralasertib Monotherapy in Patients with ATM-Altered Advanced Solid Tumors or Metastatic Castration-Resistant Prostate Cancer: Data from the Phase IIa PLANETTE Study
Source: Cancer Res Commun. 2026 Jul 2;6(7):1546–56. doi: 10.1158/2767-9764.CRC-26-0184 (PMC13324620; doi:10.1158/2767-9764.CRC-26-0184)
Supplement: Supplementary Figure 1 — CONSORT diagram for patients who started on ceralasertib 240 mg BID [file crc-26-0184_supplementary_figure_1_suppsf1.pdf]

Supplementary Figure 1. CONSORT diagram for patients who started on ceralasertib 240 mg BID

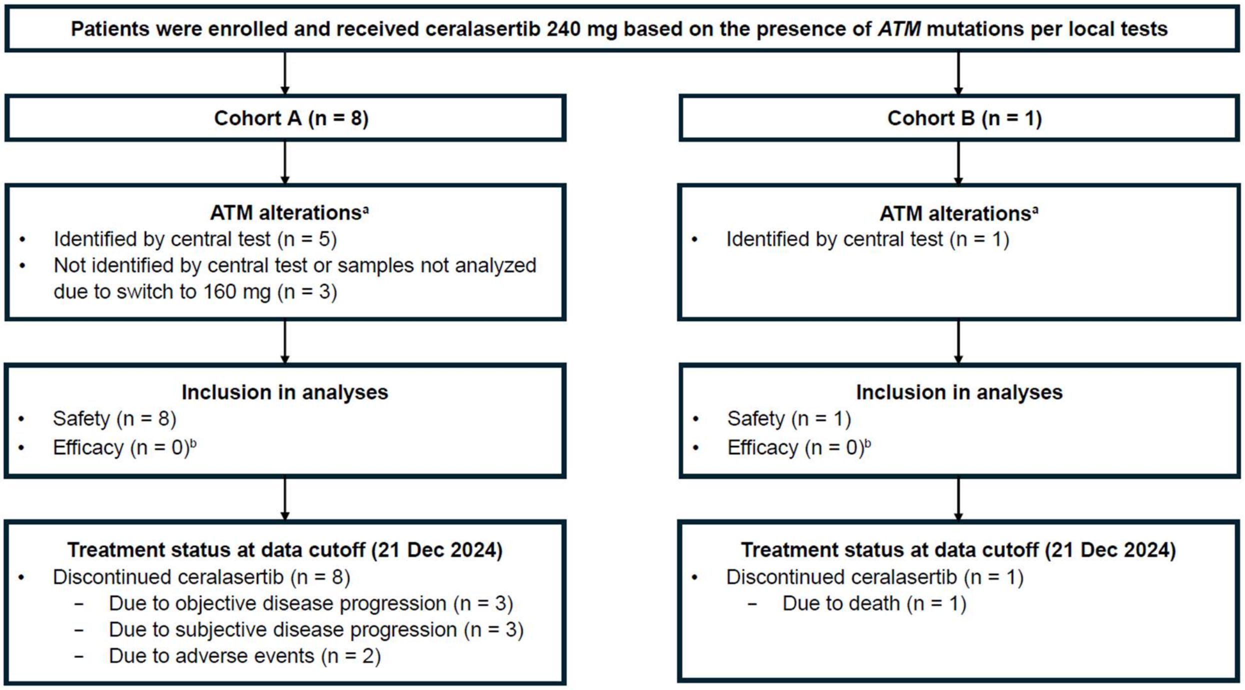

[a]*ATM* mutations and/or ATM protein loss by immunohistochemistry; [b]Efficacy analyses were not conducted due to the small sample size.

ATM, ataxia-telangiectasia mutated; BID, twice daily.
