# Supplementary material for: Ceralasertib Monotherapy in Patients with ATM-Altered Advanced Solid Tumors or Metastatic Castration-Resistant Prostate Cancer: Data from the Phase IIa PLANETTE Study
Source: Cancer Res Commun. 2026 Jul 2;6(7):1546–56. doi: 10.1158/2767-9764.CRC-26-0184 (PMC13324620; doi:10.1158/2767-9764.CRC-26-0184)
Supplement: Supplementary Figure 2 — MRI scan showing target lesion reduction in the responder with endometrial cancer receiving ceralasertib 160 mg BID in Cohort A [file crc-26-0184_supplementary_figure_2_suppsf2.pdf]

**Supplementary Figure 2.** MRI scan showing target lesion reduction in the responder with endometrial cancer receiving ceralasertib 160 mg BID in Cohort A

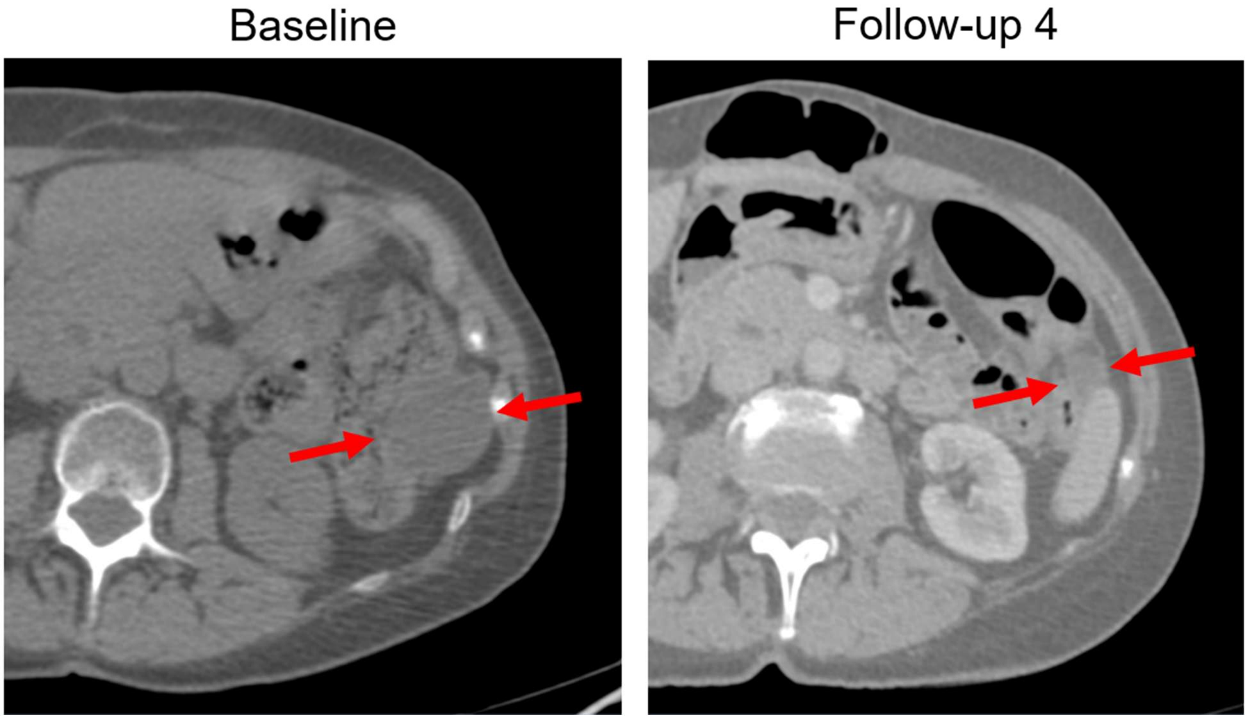


BID, twice daily; MRI, magnetic resonance imaging.
