# Supplementary material for: Ceralasertib Monotherapy in Patients with ATM-Altered Advanced Solid Tumors or Metastatic Castration-Resistant Prostate Cancer: Data from the Phase IIa PLANETTE Study
Source: Cancer Res Commun. 2026 Jul 2;6(7):1546–56. doi: 10.1158/2767-9764.CRC-26-0184 (PMC13324620; doi:10.1158/2767-9764.CRC-26-0184)
Supplement: Supplementary Figure 3 — Percentage change in PSA over time for patients with ATM alterations by central testing who started on ceralasertib 160 mg BID in Cohort B [file crc-26-0184_supplementary_figure_3_suppsf3.pdf]

Supplementary Figure 3. Percentage change in PSA over time for patients with ATM alterations by central testing who started on ceralasertib 160 mg BID in Cohort B

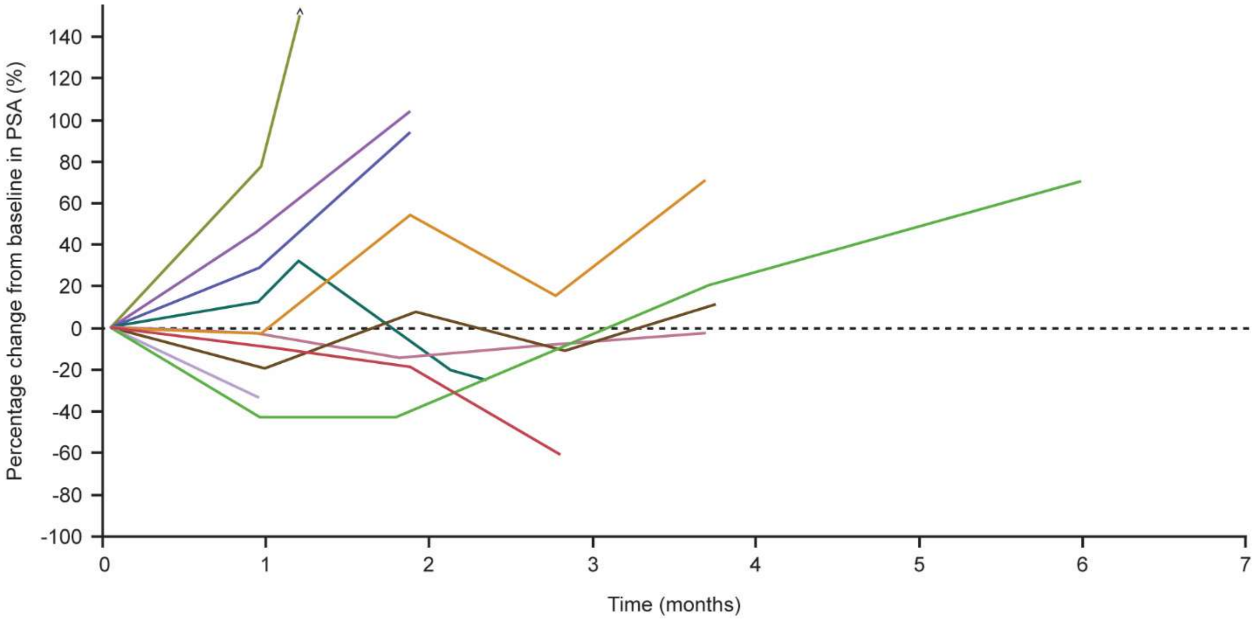

^This patient experienced an increase in PSA >680% after 2–3 months.
ATM, ataxia-telangiectasia mutated; BID, twice daily; PSA, prostate-specific antigen.
