# Supplementary material for: Ceralasertib Monotherapy in Patients with ATM-Altered Advanced Solid Tumors or Metastatic Castration-Resistant Prostate Cancer: Data from the Phase IIa PLANETTE Study
Source: Cancer Res Commun. 2026 Jul 2;6(7):1546–56. doi: 10.1158/2767-9764.CRC-26-0184 (PMC13324620; doi:10.1158/2767-9764.CRC-26-0184)
Supplement: Supplementary Figure 4 — Progression-free survival for (a) patients who started on ceralasertib 160 mg BID in Cohort A, (b) patients with ATM protein expression ≤5% versus >5% versus unknown by IHC who started on ceralasertib 160 mg BID in Cohort A, (c) patients who started on ceralasertib 160 mg BID in Cohort B, and (d) patients with ATM protein expression ≤5% versus ATM >5% versus unknown by IHC who started on ceralasertib 160 mg BID in Cohort B [file crc-26-0184_supplementary_figure_4_suppsf4.pdf]

Supplementary Figure 4. Progression-free survival for (a) patients who started on ceralasertib 160 mg BID in Cohort A, (b) patients with ATM protein expression ≤5% versus >5% versus unknown by IHC who started on ceralasertib 160 mg BID in Cohort A, (c) patients who started on ceralasertib 160 mg BID in Cohort B, and (d) patients with ATM protein expression ≤5% versus ATM >5% versus unknown by IHC who started on ceralasertib 160 mg BID in Cohort B

A

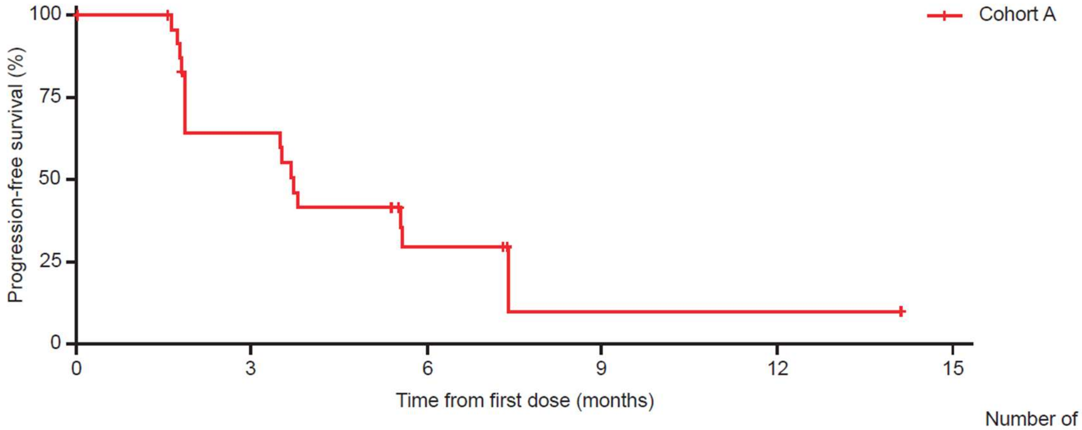

Number of subjects at risk							Number of patients treated/number of events
Cohort A	28	14	5	1	1	0	28/17

B

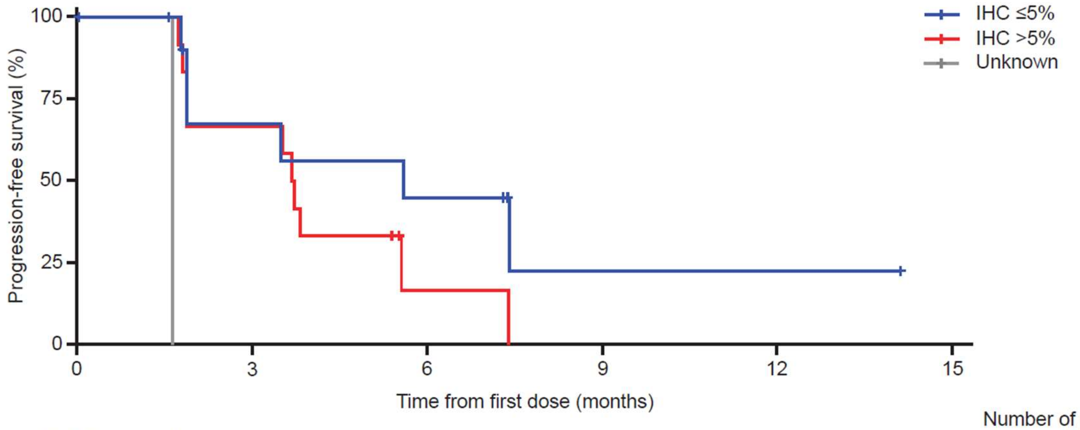

Number of subjects at risk							Number of patients treated/number of events
IHC ≤5%	11	6	4	1	1	0	11/6
IHC >5%	12	8	1	0	0	0	12/10
Unknown	5	0	0	0	0	0	5/1

C

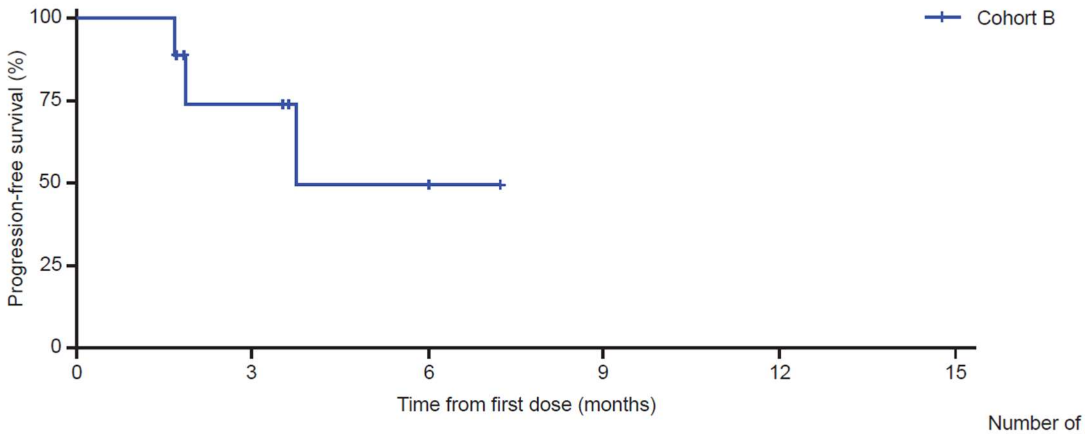

Number of subjects at risk							Number of patients treated/number of events
Cohort B	13	5	2	0	0	0	13/3

D

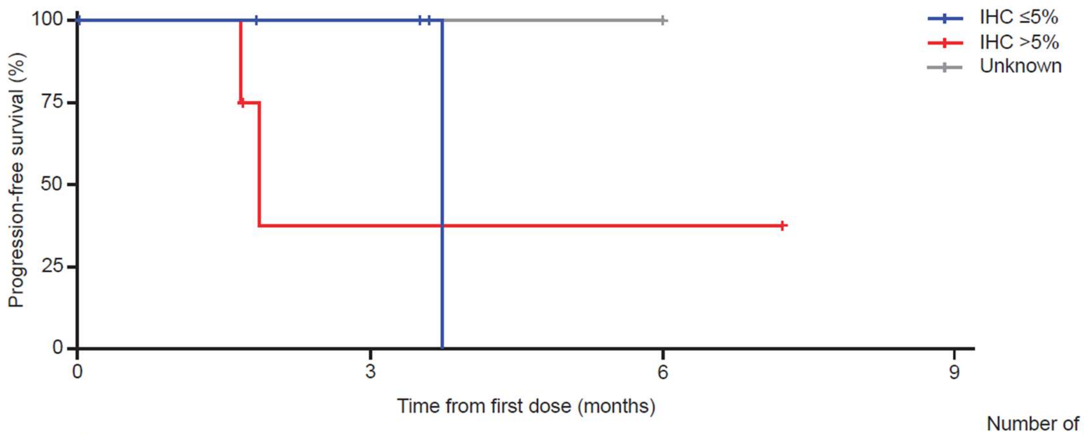

Number of subjects at risk					Number of patients treated/number of events
IHC ≤5%	7	3	0	0	7/1
IHC >5%	5	1	1	0	5/2
Unknown	1	1	1	0	1/0

ATM, ataxia-telangiectasia mutated; BID, twice daily; IHC, immunohistochemistry.
