# Supplementary material for: Ceralasertib Monotherapy in Patients with ATM-Altered Advanced Solid Tumors or Metastatic Castration-Resistant Prostate Cancer: Data from the Phase IIa PLANETTE Study
Source: Cancer Res Commun. 2026 Jul 2;6(7):1546–56. doi: 10.1158/2767-9764.CRC-26-0184 (PMC13324620; doi:10.1158/2767-9764.CRC-26-0184)
Supplement: Supplementary Figure 5 — Geometric mean plasma concentrations of ceralasertib on Day 8 and Day 14 of Cycle 1 (blue) and Cycle 2 (orange) following receipt of ceralasertib 160 mg administered BID on days 1–14 of a 28-day cycle [file crc-26-0184_supplementary_figure_5_suppsf5.pdf]

Supplementary Figure 5. Geometric mean plasma concentrations of ceralasertib on Day 8 and Day 14 of Cycle 1 (blue) and Cycle 2 (orange) following receipt of ceralasertib 160 mg administered BID on days 1–14 of a 28-day cycle

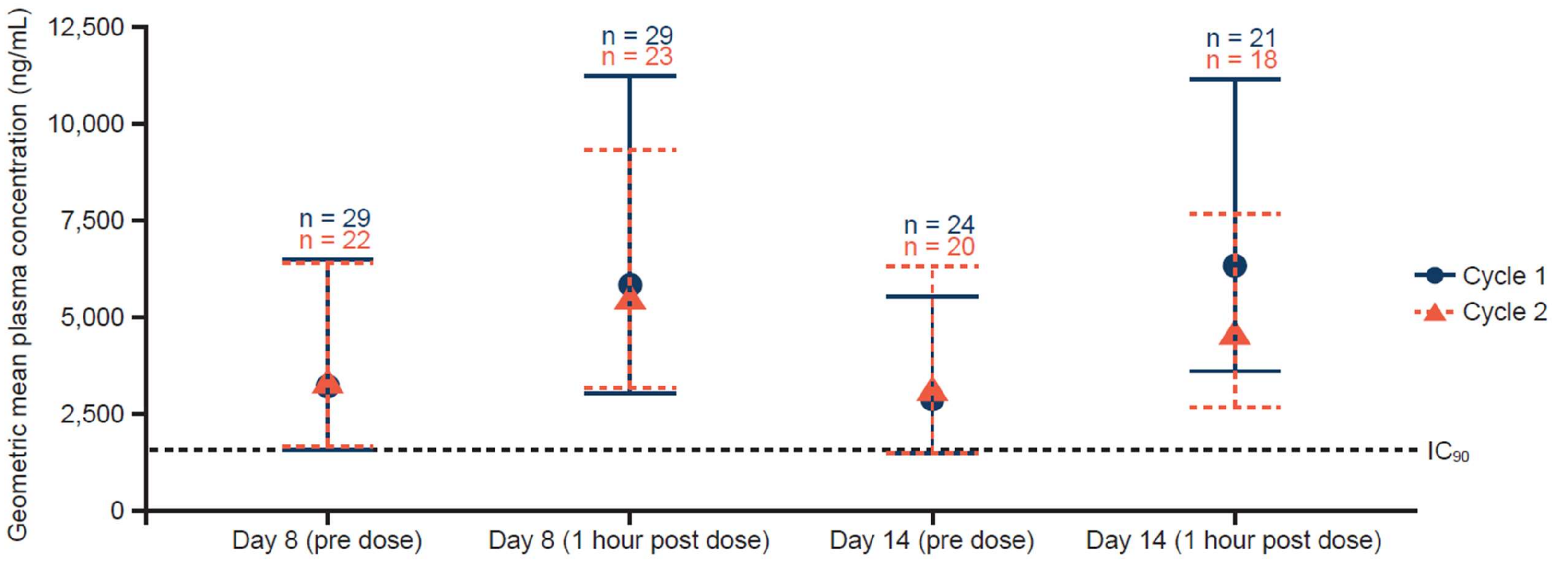

Geometric means (i.e., exp[μ], where μ is the mean of the data on the natural log scale) are represented by blue circles (Cycle 1) or orange triangles (Cycle 2). Vertical lines represent the gSD, calculated as exp(σ), where σ is the standard deviation on the natural log scale. Thus, geometric mean ± gSD = exp(mean(ln[PK concentration]) ± SD(ln[PK concentration]). N numbers indicate the number of patients with available PK data per sampling timepoint in the dataset. The dotted horizontal line shows the IC_{90}.

BID, twice daily; gSD, geometric standard deviation; PK, pharmacokinetics.
